# Molecular Characterization of Glycopeptide-Resistant Enterococci from Hospitals of the Picardy Region (France)

**DOI:** 10.1155/2010/150464

**Published:** 2010-10-31

**Authors:** M. Biendo, C. Adjidé, S. Castelain, M. Belmekki, F. Rousseau, M. Slama, O. Ganry, J. L. Schmit, F. Eb

**Affiliations:** ^1^Service de Bactériologie, CHU Nord, Place Victor Pauchet, 80054 Amiens Cedex 1, France; ^2^Service d'Epidémiologie, Hygiène Hospitalière et Santé Publique, CHU Nord, Place Victor Pauchet, 80054 Amiens Cedex 1, France; ^3^Unité de Virologie clinique et fondamentale, Faculté de Médecine et de Pharmacie, 3 rue des Louvels, 80036 Amiens, Cedex, France; ^4^Service de Réanimation Néphrologique, CHU Sud, avenue René Laënnec, 80054 Amiens Cedex 1, France; ^5^ Service de Pathologie Infectieuse, CHU Nord, Place Victor Pauchet, 80054 Amiens Cedex 1, France

## Abstract

We studied 138 glycopeptide-resistant enterococci (GRE) strains, consisting of 131 glycopeptide-resistant *Enterococcus faecium* (GREfm) and 7 glycopeptide-resistant *Enterococcus faecalis* (GREfs). The GREfm strains were resistant to penicillin, ampicillin, vancomycin, and teicoplanin, while the GREfs strains were only resistant to vancomycin and teicoplanin. The *van A* gene was the only glycopeptide determinant present in all GRE isolates investigated. Genes coding for Hyl and Hyl+ Esp were detected in 39 (29.8%) and 92 (70.2%) of the 131 GREfm isolates, respectively. Three of the 7 GREfs were positive for *gelE*+*asa 1* genes, 3 for *gel E* gene, and 1 for *asa 1* gene. The genetic relationship between the 138 GRE was analyzed by pulsed-field gel electrophoresis (PFGE) and multilocus sequence typing (MLST). GREfm isolates were clustered in a single genogroup (pulsotype A), and GREfs were clustered in six genogroups (pulsotypes B-G). Among the isolates investigated by MLST, only 18 PCR products were sequenced (12 *E. faecium* and 6 *E. faecalis*), and 9 sequence types (STs) were identified.

## 1. Introduction

Enterococci form part of the normal flora of both the human and animal gastrointestinal tract but are also found in other anatomical sites including the vagina and oral cavity. Of the 20 enterococcal species known, *Enterococcus faecalis* and *Enterococcus faecium* are among the leading causes of several human infections, including bacteremia, septicemia, endocarditis, urinary tract infections, wound infections, neonatal sepsis, and meningitidis.

Glycopeptide-resistant enterococci (GRE) are a mutant of *Enterococcus* that originally developed in individuals exposed to antibiotics. They have increasingly emerged as a major cause of nosocomial infections worldwide [1]. This emergence has been associated with gradual replacement of *Enterococcus faecalis* by *Enterococcus faecium* and an epidemic rise of vancomycin-resistant *E. faecium* [2]. Vancomycin is the antibiotic of choice for infections caused by penicillin-resistant strains, alone or in combination with aminoglycosides. Acquired vancomycin resistance to this organism greatly reduces the number of treatment options and, therefore, constitutes a major therapeutic concern. This problem is further compounded by the fact that resistance genes can potentially be transferred to other pathogenic organisms such as *Staphylococcus aureus*.

GRE strains were reported for the first time in France and the United Kingdom in 1988 [3], and then in the USA [4]. In France, the incidence of glycopeptide resistance in *E. faecium* bacteremia is less than 5% [3], the proportion of GRE is less than 2%, and the prevalence rate has remained at 0.01% [5, 6].

The main risk factor for the development of GRE strains is the excessive use of glycopeptides, but the use of third-generation cephalosporins and fluoroquinolones is also involved in the selection of GRE [7].

Three glycopeptide resistance phenotypes have been distinguished in the GRE strains on the basis of the level and inducibility of resistance to vancomycin and teicoplanin [8]. The Van A type is characterized by acquired inducible resistance to both vancomycin and teicoplanin [9]. Strains of the Van B type have acquired inducible resistance to various levels of vancomycin but not to teicoplanin [10]. Constitutive low-level resistance to vancomycin (Van C1, Van C2/3, Van E, and Van G phenotypes) is an intrinsic property of motile enterococci, *E. gallinarum*, *E. casseliflavus*, and *E. flavescens* [11, 12]. *van A *and* van B* are the main resistance genotypes reported for *E. faecalis* and *E. faecium*, the two species most frequently isolated from clinical sites. Numerous factors are associated with a greater risk of acquiring enterococcal infections. These factors, including antimicrobial resistance and expression of virulence factors associated with infection-derived *E. faecalis* and *E. faecium* strains, possess several putative virulence determinants, including aggregation substance, gelatinase, cytolysin, enterococcal surface protein, and hyaluronidase [13]. The first four virulence factors are found in *E. faecalis*, while the fourth and fifth virulence factors are specific for *E. faecium* [13].

Aggregation substance, encoded by *asa1*, which is carried on a plasmid, is a pheromone-inducible protein that enables the conjugative transfer of sex pheromone gene-containing plasmids through the clumping of one *Enterococcus* to another [14]. As a virulence factor, aggregation substance increases bacterial adherence to renal tubular cells [15] and heart endocardial cells [16].

Gelatinase, encoded by the chromosomal *gelE*, is an extra cellular zinc endopeptidase that hydrolyzes collagen, gelatine, and small peptides [17].

The production of cytolysin has also been shown to significantly worsen the severity of endocarditis [18]. Cytolysin genes are carried on a plasmid or are integrated into the bacterial chromosome [19]. Cytolysin consists of two components, lysine (L) and activator (A). The cytolysin operon consists of five genes, of which *cyl L1*, * cyl L2, cyl M, *and *cyl B* are relevant to the expression of component L, whereas *cyl A* is necessary for the expression of component A.

The enterococcal surface protein, encoded by the chromosomal gene *esp*, has an interesting structure that includes a central core consisting of distinct tandem repeat units. Enterococcal surface protein is associated with increased virulence [20], colonization and persistence in the urinary tract, and biofilm formation [21].

Another virulence factor, hyaluronidase, was described in *E. faecium* [22]. The *E. faecium* hyaluronidase, encoded by the chromosomal gene *hyl, *shows homology to the hyaluronidases previously described in *Streptococcus pyogenes*, *Staphylococcus aureus*, and *Streptococcus pneumoniae* which are believed to contribute to invasion of the nasopharynx and pneumococcal pneumonia [23].

The aim of this study was to use pulsed-field gel electrophoresis (PFGE) and multilocus sequence typing (MLST) to characterize glycopeptide-resistant *E. faecium* (GREfm) and glycopeptide-resistant *E. faecalis* (GREfs) isolates from clinical specimens obtained from patients admitted to Picardy hospitals (France). The *van* genotypes of the GRE isolates were determined, and the virulence factor genes were detected.

## 2. Materials and Methods

### 2.1. Setting

One hundred thirty-eight GRE clinical isolates obtained from 127 patients were collected from five hospitals in the Picardy region (Amiens University Hospital (AUH; 128 isolates), Picardy Private Hospital (PPH; 6 isolates), Montdidier hospital (MH; 2 isolates), Doullens Hospital (DH; 1 isolate), and Saint Quentin Hospital (SQH; 1 isolate)) between April 2004 and January 2009. Clinical isolates were recovered from rectal swabs (*n* = 103), from urine (*n* = 12), from pus (*n* = 6), from peritoneal fluid (*n* = 4), from blood (*n* = 3), from drainage tube (*n* = 2), from bile (*n* = 2), from urethral swab (*n* = 1), from vaginal swab (*n* = 1), from abscess (*n* = 1), from catheter (*n* = 1), from pyosalpinx (*n* = 1), and from sputum (*n* = 1) (Table 1).

The medical records of all patients with GRE isolates were reviewed retrospectively. Clinical data collected included age, gender, the hospital and the ward in which these patients were hospitalized and where they came.

### 2.2. Defining Samples

In this study, the clinical samples are the samples taken for diagnosis, and the rectal swabs are the samples taken for screening.

### 2.3. Culture and Phenotypic Identification

Rectal swabs were cultured on Enterococcosel selective agar supplemented with 8 *μ*g/mL vancomycin (Becton Dickinson, France) with teicoplanin disc. Clinical samples were cultured on Columbia agar supplemented with 5% defibrinated horse blood, and both were incubated aerobically for 24–48 hours at 37°C. Isolates were identified as *E. faecium* or *E. faecalis* by rapid ID32 Strep system according to the manufacturer's instructions (BioMérieux, France).

### 2.4. Antimicrobial Susceptibility Testing

Resistance to vancomycin, teicoplanin, penicillin, and ampicillin and to kanamycin, gentamicin, and streptomycin was tested by disc diffusion methods according to the *Comité de l'Antibiogramme de la Société Française de Microbiologie* (CA-SFM) guidelines [24]. Minimum inhibitory concentrations (MICs) of these antimicrobial agents were determined using E-test strips (BioMérieux, France), and the results were interpreted according to established breakpoint values [24]. *E. faecium* Van A CIP 104676 and *E. faecalis* Van B CIP 104105 standard strains were used as reference strains.

### 2.5. Identification of GRE and Glycopeptide Resistance Determinants

Total DNA was extracted from enterococci by using the BioRobot EZ1 extractor apparatus (QIAGEN, France) according to the manufacturer's recommendations. In order to determine the genotype responsible for glycopeptide resistant strains, we used a multiplex PCR (mPCR) as previously described [25]. During the mPCR, the DNA fragments were identified according to their size. This mPCR allowed the simultaneous detection of glycopeptide resistance genotypes: *van A, van B, van C1,* and *van C2/3* (Table 2). PCR was performed on a DNA thermal cycler (model MJ, MINI Gradient, BioRad, France) in a final volume of 50 *μ*L containing 25 *μ*L GoTaq Green Master Mix (Promega, USA), 20 pmol of each oligonucleotide primer pair, and 1 *μ*L of DNA as template. The cycling conditions were 94°C for 2 minutes followed by 30 cycles of 94°C for 1 minute, 54°C for 1 minute, 72°C for 1 minute, and then 72°C for 10 minutes. PCR products were resolved by electrophoresis on a 1% agarose-Tris-EDTA gel containing 0.5 *μ*g per mL of ethidium bromide. Smartladder (Eurogentec, Belgium) was used as molecular weight marker.

After the PCR test, the PCR products obtained were hybridized with membrane strips coated with *E. faecium, E. faecalis, E. gallinarum,* and *E. casseliflavus* species using the specific probes provided with the Genotype *Enterococcus* kit (Hain Lifescience GmbH, Germany). The hybridization procedures were performed according to the manufacturer's recommendations. This technique was used to confirm combined identification of enterococcal species and *van* resistance genes.

### 2.6. Detection of Genes Encoding GRE Virulence Factors by Multiplex PCR

The presence of five genes encoding virulence factors in GRE isolates [14, 22, 26] were investigated by multiplex PCR using the oligonucleotide primer pairs previously reported [10] (Table 3), for which primers were designed based on published DNA sequences from the GenBank database. The choice of these five genes was based, for which they constitute the principal virulence factor genes reported for *E. faecalis* and *E. faecium *strains, on the high frequency reported in Europe [3] and in France [27] in enterococci strains, and on their use in mPCR schemes [13]. PCR was performed as described above. Each 50 *μ*L PCR mixture consisted of 25 *μ*L Gotaq Green Master Mix, 20 pmol of each oligonucleotide primer pair for *asa1, gel E, hyl, cyl A,* and *esp*, and 5 *μ*L of DNA as template. Amplifications were performed under the following conditions: 95°C for 15 minutes, followed by 30 cycles of 1 minute at 94°C, 1 minute at 56°C, 1 minute at 72°C, and then 10 minutes at 72°C for the last cycle. PCR products were then sequenced.

### 2.7. PFGE

Macrorestriction analysis by PFGE was performed for 131 *E. faecium *and 7 *E. faecalis* isolates with *SmaI*-restricted whole-cell DNA embedded in 1% agarose plugs and separated in a 1.2% pulsed-certified agarose gel with a contour-clamped Homogeneous Electric Field (CHEF DRII apparatus; BioRad, France). *E. faecium* Van A CIP 104676 and *E. faecalis* Van B CIP 104105 strains were used as reference strains. Concatemers of bacteriophage *λ*-ladder were used as molecular weight marker (BioRad, France). PFGE patterns were interpreted according to the criteria of Tenover et al. [28]. The similarity dendrogram was constructed with the unweighted pair-group method with arithmetic means (UPGMA) using the DICE correlation coefficient.

### 2.8. MLST Sequence Types

Four housekeeping genes (loci) (Table 4) were amplified for each isolate [29]. The choice of these housekeeping genes was based on their putative function and on their use in MLST schemes of *E. faecium.* Information on these loci is available at the MLST web site (http://efaecium-mslt.net).

### 2.9. PCR

Internal 400- to 600-bp gene fragments were amplified by PCR. Reactions were performed in 50 *μ*L volumes composed of 25 *μ*L GoTaq Green Master Mix, 20 pmol of each oligonucleotide primer pair, and 1 *μ*L of bacterial DNA as template. PCR conditions for all amplification reactions were as follows: 94°C for 3 minutes, 35 cycles at 94°C for 30 seconds, 50°C for 30 seconds, 72°C for 30 seconds, and 72°C for 5 minutes The amplicons were analyzed by electrophoresis as described above.

### 2.10. MLST Data Analysis

Sequences of each allele were trimmed and compared with all alleles in the MLST database. Each unique nucleotide sequence was assigned a unique allele number. The allele profile for each isolate was determined and consisted of a line listing of allele numbers for each gene. Isolates were then assigned a sequence type (ST) according to their allelic profiles.

### 2.11. Nucleotide Sequence Accession Numbers

The sequences of *van* genes, virulence factor genes, and the alleles housekeeping genes have been given the following GenBank accession numbers: HM641733 (*van A*), HM 641734 (*asa1*), HM 641735 (*gel E*), HM 641736 (*psts*), HM 641737 (*gdh*), and HM 641738 (*gyd*) for *E. faecalis*; HM 641739 (*van A*), HM 641740 (*hyl*), HM 641741 (*esp*), HM 641742 (*psts*), HM 641743 (*gdh*), HM 641744 (*gyd*), and HM 641745 (*esp*) for *E. faecium*.

## 3. Results

### 3.1. Epidemiology

GRE was isolated from a total of 127 patients hospitalized during the period from April 2004 through January 2009. The first GRE isolates observed were two *E. faecalis* strains isolated from urinary tract infection of patients hospitalized in the AUH nephrology and orthopedic wards (April and May 2004, resp.). From 2005 to 2008, five other GREfs were isolated (three from rectal swabs and two from vaginal swab and pus) from patients hospitalized in AUH wards.

From July to November 2005, during an outbreak comprising a limited number of cases, 33 GREfm (23 from rectal swabs and 10 from peritoneal fluid, blood, drainage tube, pus, bile, and catheter) were obtained from 25 colonized/infected patients, also hospitalized in AUH wards.

The major outbreak occurred from May 2006 to January 2009. Ninety-eight GREfm (22 isolates from clinical samples and 76 isolates from screening rectal swabs) were obtained from 95 patients hospitalized at AUH (85 patients), PPH (6 patients), MH (2 patients), DH (1 patient), and SQH (1 patient). The most recent isolates were obtained during fecal screening of all hospitalized contact patients, as part of infection control measures.

### 3.2. Patients Carrying GRE Isolates

Of 127 hospitalized patients included in this study, 67 (52.7%) were men and 60 (47.3%) were women. The mean age of these patients was 70.1 years (range: 19–98 years) in men and 73.6 years (range: 16–95 years) in women (M/F sex ratio: 1 : 12). These patients were classified as either colonized, 74.8% (95/127), or infected, 25.2% (32/127), according to the definitions of French guidelines [30] based on those of the Centers for Disease Control and Prevention [31]. This distribution confirms the low ratio of infections versus colonization. The patient distribution according to GRE-positive specimens showed that 94 patients had one GRE-positive surveillance rectal swab and 1 patient had 3 GRE-positive surveillance rectal swabs; twenty-four patients had one GRE-positive clinical sample, and 8 patients had 11 GRE-positive clinical samples plus 6 GRE-positive surveillance rectal swabs. Application of infection control measures included weekly surveillance cultures and environmental decontamination guided by culture and PCR-hybridization results.

### 3.3. PCR Assays and Sequencing Results

Sequencing yielded five distinct DNA sequences: one from Van A PCR (732 bp), one from Esp (510 bp), one from Asa 1 (375 bp), one from Hyl (276 bp), and one from Gel E (213 bp). The nucleotide and amino acid sequences of *van *gene and virulence factor genes obtained were compared to the known sequences of Genbank. The nucleotide and amino acid sequences of the *van A* gene had 100% genetic identity and 100% amino acid identity with *E. faecium* pIP816 plasmid, accession n°AM932524. The *esp* gene sequences exhibited a 100% nucleotide and amino acid homology with sequences for *E. faecium *isolate E470 putative enterococcal surface protein (*esp*) gene, accession n°AY322500. The nucleotide and amino acid sequences of *asa1* gene from our *E. faecalis* strains were identical to those known of *E. faecalis* plasmid pAD1 *asa1* gene for aggregation substance (100% genetic and amino acid identity), accession n°X17214. The Hyl sequences showed best similarity with *E. faecium* putative hyaluronidase (*hyl*) gene (100% genetic identity; 100% amino acid identity), accession n°AF544400, while the partial *Gel E* sequences showed best similarity with *E. faecalis* gelatinase (*gel E*) gene (100% genetic identity; 100% amino acid identity), accession n°M37185.

### 3.4. Molecular Identification, Antibiotic Susceptibility, and Virulence Factors

The molecular identification of 138 GRE showed that 131 enterococci isolates belonged to the *E. faecium* species (94.9%) and 7 (5.1%) belonged to the *E. faecalis* species. The *van A* gene was the only glycopeptide resistance determinant found in all isolates studied. The resistance patterns for the isolates are shown in Table 5. The 131 GREfm isolates were resistant to penicillin (MICs, 96 to >256 *μ*g/mL) and ampicillin (MICs, 48 to >256 *μ*g/mL). One hundred twenty-eight of these isolates showed HLKR [(high-level kanamycin resistance) (MICs, >256 to >512 *μ*g/mL)], 116 showed HLGR [(high-level gentamicin resistance) (MICs, >256 to >512 *μ*g/mL)], and 53 showed HLSR [(high-level streptomycin resistance) (MICs, >256 to >512 *μ*g/mL)]. The 7 GREfs were susceptible to penicillin (MICs, 1.5–4 *μ*g/mL) and ampicillin (MICs, 0.50–1.5 *μ*g/mL). Six isolates showed HLKR (MICs of >512 *μ*g/mL) and HLGR (MICs, >256 to >512 *μ*g/mL), and one isolate showed HLSR (MIC, >512 *μ*g/mL). Glycopeptide susceptibility test results were in agreement with resistance genotypes: the MICs of vancomycin were > 256 *μ*g/mL, and the MICs of teicoplanin were 32 to >256 *μ*g/mL. Genes coding for Hyl and Hyl+ Esp were detected in 39 (29.8%) and 92 (70.2%) of the 131 GREfm isolates, respectively. Three of the 7 GREfs were positive for *gel E*+ *asa 1* genes, 3 were positive for *gel E* gene, and 1 was positive for *asa 1* gene (Table 5). The *cyl A* gene was not detected in any of the GRE isolates examined.

### 3.5. Molecular Typing and Clonal Characteristics of GRE

Analysis of PFGE profiles showed that 131 GREfm isolates shared a similar electrophoretic profile, designated type A, and were clonally related. This PFGE type A encompassed 26 different subtypes (A1–A26). Subtypes A16 and A26 each corresponded to two isolates. The other subtypes corresponded to single isolate. The seven GREfs isolates appeared to be more heterogeneous on the basis of their PFGE profiles in six different types (B–G). Only type G was identified in two isolates with identical profiles. These two isolates were obtained from two different patients hospitalized in different wards. These pulsotypes were considered to be sporadic profiles [32].

### 3.6. Diversity of Housekeeping Genes

MLST PCR was performed for all 138 isolates belonging to PFGE types A–G, but only 18 PCR products were sequenced. These 18 PCR products were selected as being representative of all anatomical sites of sampled patients and all hospital clinical wards during the study period. Twelve GREfm isolates were selected because they shared the same PFGE type A and subtypes (A1, A3, A5–A7, A10, A15, A16, A20, and A26) and were isolated at various times over the study period. The six GREfs isolates were chosen because of their different PFGE profiles (B–G) showing a difference of at least six bands from PFGE pattern A [33].

**Figure 1:**
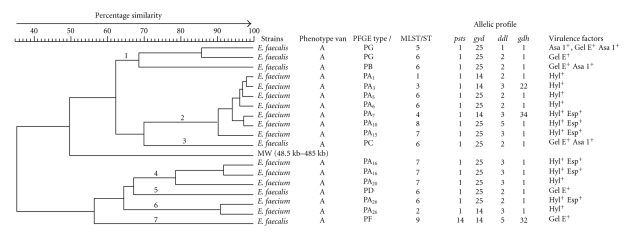
PFGE dendrogram with the corresponding MLST sequence types of the GRE isolates, digested with *SmaI*. Strains were clustered by the unweighted pair-group method with arithmetic mean (UPGMA). Each row represents a unique PFGE type with its unique PFGE pattern. The corresponding MLST sequence type (ST) is shown with the corresponding strains. Molecular weight: (MW). Bacteriophage *λ*-ladder is given in kbp (48.5 kb–485.0 kb) *PFGE type A (PA) isolates 3, 4, 6–8, 10–13–24, 27, 29–32, 34, 36, 37, 39–43, 45, 46, 49–53, 55–60, 63–101, 103–115, 117–138. (+): positive.

The restriction profiles of GREfm strains, presented in the dendrogram (Figure 1), show that the 12 selected pulsotype A strains belong to subtypes A1, A3, A5–A7, A10, A15, A16, A20 and A26. These subtypes correspond to 3 clones. The clone 2 included 7 strains belonging to subtypes A1, A3, A5, A6, A7, A10, and A15. The percentage of similarity between each strain was between 90% and 98%. The 3 strains of clone 4 belong to subtypes A16 and A20 and present the percentage of similarity between 78% and 91%. Finally, the 2 strains of clone 6 belong to subtype A26 and have a percentage of similarity of 90%. In total, the 12 subtypes A presenting a percentage of similarity between 78% and 98% show the propagation of one GREfm clone in hospitals of the Picardy region. The 6 GREfs pulsotypes analysis allowed to identify 4 clones. Clone 1 included 3 strains which present a percentage of similarity between 70% and 86%, two of these strains belonging to pulsotype G have 86% of homology and the remaining one strain belonging to pulsotype B has 70% of homology. The strains of clones 3,5, and 7 belonging to pulsotypes C, D, and F have 72%, 67%, and 57% of homology, respectively. These strains were isolated from patients hospitalized at nephrology, orthopedic surgery, thoracic surgery, and endocrinology wards and two patients from hepatology and gastroenterology wards. The dendrogram (Figure 1) may let us fear the emergence of other ERGfs clones as well as the dispersion of these bacteria following an epidemic mode, as seems to be the case of clone 1.

MLST analysis of the 18 isolates revealed 9 STs (Figure 1). The sequence types most frequently identified were ST6 (7 isolates) and ST7 (4 isolates) which shared the four housekeeping alleles, while ST1 (1 isolate), ST2 (1 isolate), ST3 (1 isolate), ST4 (1 isolate), ST5 (1 isolate), and ST8 (1 isolate) shared three of the four housekeeping alleles. The majority of these isolates belonged to the *E. faecium* species. They were clustered together with PFGE and were, therefore, considered to belong to the same pulsotype A. They were involved in a sustained outbreak in the hospitals of the Picardy region. Of six *E. faecalis* isolates, five were genotypically different and corresponded to 3 different STs (ST5, ST6, and ST9) (Figure 1). These STs differed from each other at one or four loci.

## 4. Discussion

GRE are distributed worldwide, but their epidemiology appears to vary on a regional basis. Thus, polyclonal isolates were described [34], whereas some European Centres have reported nosocomial outbreak of GRE associated with very diverse epidemiological situations [3]. The data presented in this study show that most of the hospital-derived GREfm are part of a single clonal (pulsotype A). Among the acquired genes conferring resistance to glycopeptides, *van A* is the only identified determinant. The use of multiplex PCR allowed simultaneous detection of enterococcal genes that encode for aggregation substance (asa 1), gelatinase (gel E), cytolysin (cyl A), enterococcal surface protein (esp), and hyaluronidase (hyl). In 131 GREfm isolates, the *asa 1* and *gel E *genes were not detected in this study which is in agreement with the results reported by other investigators [13, 35]. In contrast, these genes were found in GREfs. 

The combined presence of *hyl* and *esp* genes was found in 70.2% of 131 GREfm isolates tested, which is in accordance with the findings of Vankerckhoven et al. [13] and Rice et al. [22]. In contrast, the only *esp *gene was not detected, as described elsewhere [13, 36]. The *hyl* gene was found in 29.8% of the 131 GREfm isolates, which is in contrast with the findings of Vankerckhoven et al. [13], who detected *hyl* gene in only 17% of the *E. faecium *isolates collected.

PFGE has been proposed as the method of choice from epidemiological typing of GREfm [37], although alternative technique, such as MLST analysis, has also been used successfully for characterization of GRE isolates [33]. The findings obtained by PFGE regarding the clonality of GREfm isolates were in accordance with MLST typing. A subpopulation of *E. faecium* adapted to the hospital setting, and consisting of strains responsible for epidemics, was characterized. The 12 representative GREfm isolates of pulsotype A analyzed by MLST belonged to ST1–ST4, ST6–ST8. These isolates are characterized by the presence of Hyl and Esp virulence factors as GREfm markers and high-level resistance to penicillin, ampicillin, vancomycin, and teicoplanin, in accordance with data of the literature [38, 39]. Top et al. [40] showed that their epidemic strains belonging to certain STs have been grouped in clonal complex 17 (CC17) of *E. faecium.* CC17 was defined upon MLST and is characterized by resistance to quinolones and ampicillin and the presence of the enterococcal surface protein (Esp) in the majority of isolates. The Hyl and Esp virulence factors have also been detected in vancomycin-susceptible strains [10]. The hypothesis proposed to explain the widespread distribution is the emergence of epidemic strains adapted to the hospital setting, which acquired vancomycin resistance determinants by horizontal gene transfer.

In this study, PFGE demonstrated the existence of a GREfm epidemic clone within the *E. faecium* population. The allelic profiles of this clone are relatively homogeneous, which suggests that they are genetically related. The GREfs isolates investigated by MLST were grouped into six different PFGE genogroups. This genetic diversity may not have emerged in the *E. faecium *epidemic population.

In conclusion, our data indicate that GREfm van A strains remain predominant in our region among GRE isolates, unlike that of the next North region of France, where an *E. faecium vanB* outbreak has been reported [6]. The recent increase in the number of GREfm in hospitals of the Picardy region might be due to the appearance and spread of a hospital-adapted, multidrug-resistant GREfm clone belonging to an internationally disseminated lineage. Horizontal gene transfer and clonal spread may both have contributed to the high rate of GREfm colonizations/infections. The divisions of colonized/infected patients into sectors, and an increased surveillance during the rehospitalizations of these patients, allow for the circumscription of the epidemic.

## Figures and Tables

**Table 1:** Frequency of glycopeptide-resistant enterococci (GRE) in relation to the number of clinical samples.

Source	Specimen	Absolute number of GRE isolates	Relative frequency (%)
Hospital surveillance	Rectal swabs	103	74.7

Hospital clinical samples (*n* = 35)	Urine	12	8.8
	Pus	6	4.4
	Peritoneal fluid	4	2.9
	Blood	3	2.2
	Drainage tube	2	1.4
	Bile	2	1.4
	Urethral swab	1	0.7
	Vaginal swab	1	0.7
	Abscess	1	0.7
	Catheter	1	0.7
	Pyosalpinx	1	0.7
	Sputum	1	0.7

Total		138	100.0

**Table 2:** Oligonucleotide primers used to amplify *van* genes.

Amplified gene	Oligonucleotide sequence (5′ → 3′)	Position	PCR product size (bp)
*van A*	A_1_-5′-GGG-AAA-ACG-ACA-ATT-GC-3′	175–191	732
	A_2_-5′-GTA-CAA-TGC-GGC-CGT-TA-3′	907–891	
*van B*	B_1_-5′-ATG-GGA-AGC-CGA-TAG-TC-3′	173–189	635
	B_2_-5′-GAT-TTC-GTT-CTT-CGA-CC-3′	807–791	
*van C-1*	C_1_-5′-GGT-ATC-AAG-GAA-ACC-TC-3′	246–272	822
	C_2_-5′-CTT-CCG-CCA-TCA-TAG-CT-3′	1067–1051	
*van C-2, van C-3*	D_1_-5′-CTC-CTA-CGA-TTC-TCT-TG-3′	455–486	439
	D_2_-5′-CGA-GCA-AGA-CCT-TTA-AG-3′	885–869	

**Table 3:** Oligonucleotide primers used to amplify virulence factor genes.

Gene	Virulence factor	Primer name	Oligonucleotide sequence (5′ to 3′)	Product size (bp)
*asa1*	Aggregation substance, encoded by the plasmid *asa1 *	ASA 11	GCA-CGC-TAT-TAC- GAA -CTA-TGA	375
		ASA 12	TAA-GAA-AGA-ACA-TCA-CCA-CGA	
*gelE*	Gelatinase, encoded by the chromosomal *gelE *	GEL 11	TAT-GAC-AAT-GCT-TTT-TGG-GAT	213
		GEL 12	AGA-TGC-ACC-CGA-AAT-AAT-ATA	
*cylA*	Cytolysin, encoded by the plasmid *cylA *	CYT I	ACT-CGG-GGA-TTG-ATA-GGC	688
		CYT II	GCT-GCT-AAA-GCT-GCG-CTT	
*esp*	Enterococcal surface protein, encoded by the chromosomal *esp *	ESP 14F	AGA-TTT-CAT-CTT-TGA-TTC-TTG-G	510
		ESP 12R	AAT-TGA-TTC-TTT-AGC-ATC-TGG	
*hyl*	Hyaluronidase, encoded by the chromosomal *hyl *	HYL n1	ACA-GAA-GAG-CTG-CAG-GAA-ATG	276
		HYL n2	GAC-TGA-CGT-CCA-AGT-TTC-CAA	

**Table 4:** Amplification and sequencing primers for* ddl, gyd, gdh, *and* psts. *

Gene	Primer name	Oligonucleotide sequence (5′ to 3′)	Product size (bp)
*ddl*	DDL1	GAG-ACA-TTG-AAT-ATG-CCT-TAT-G	465
	DDL2	AAA-AAG-AAA-TCG-CAC-CG	
*gdh*	GDH1	GGC-GCA-CTA-AAA-GAT-ATG-GT	530
	GDH2	CCA-AGA-TTG-GGC-AAC-TTC-GTC-CCA	
*gyd*	GYD1	CAA-ACT-GCT-TAG-CTC-CAA-GGC	556
	GYD2	CAT-TTC-GTT-GTC-ATA-CCA-AGC	
*psts*	PSTS1	TTG-AGC-CAA-GTC-GAA-GCT-GGA	583
	PSTS2	CGT-GAT-CAC-GTT-CTA-CTT-CC	

*ddl:* D-alanine-D-alanine ligase, *gdh:* glucose-6-phosphate dehydrogenase, *gyd:* glyceraldehyde-3-phosphate dehydrogenase, and *psts: *phosphate ATP-binding cassette transporter.

**Table 5:** Characteristics of glycopeptide-resistant isolates.

Isolate type	Phenotype Van	Susceptibility data (MIC-*μ*g/mL)				PFGE type	Virulence factors
		Penicillin	Ampicillin	Vancomycin	Teicoplanin		
	A	3	1.5	>256	32	B	Gel E^+^ Asa1^+^
GRE* faecalis * *n* = 7	A	3	1.5	>256	32	C	Gel E^+^ Asa1^+^
	A	3	0.50	>256	32	D	Gel E^+^
	A	3	0.75	>256	32	E	Gel E^+^
	A	1.5	1.5	>256	>256	F	Gel E^+^
	A	2	1.5	>256	32	G	Asa1^+^
	A	4	1	>256	32	G	Gel E^+^ Asa 1^+^

GRE* faecium *	39 (29.8%) A	96 to >256	48 to >256	>256	32 to >256	A	Hyl^+^
*n* = 131	92 (70.2%) A	96 to >256	48 to >256	>256	32 to >256	A	Hyl^+^ Esp^+^

Asa1: Aggregation substance; gel E: Gelatinase, cylA: Cytolysin; esp: Enterococcal surface protein; hyl: Hyaluronidase.
